# Effects of Subcortical Atrophy and Alzheimer’s Pathology on Cognition in Elderly Type 2 Diabetes: The Alzheimer’s Disease Neuroimaging Initiative Study

**DOI:** 10.3389/fnagi.2022.781938

**Published:** 2022-01-31

**Authors:** Wen Zhang, Jiaming Lu, Zhao Qing, Xin Zhang, Hui Zhao, Yan Bi, Bing Zhang, the Alzheimer’s Disease Neuroimaging Initiative

**Affiliations:** ^1^Department of Radiology, Nanjing Drum Tower Hospital, The Affiliated Hospital of Nanjing University Medical School, Nanjing, China; ^2^Department of Neurology, Nanjing Drum Tower Hospital, The Affiliated Hospital of Nanjing University Medical School, Nanjing, China; ^3^Department of Endocrinology, Nanjing Drum Tower Hospital, The Affiliated Hospital of Nanjing University Medical School, Nanjing, China

**Keywords:** type 2 diabetes, subcortical atrophy, Alzheimer’s disease (AD), amyloid-β (Aβ), cognition

## Abstract

**Background:**

Subcortical atrophy and increased cerebral β-amyloid and tau deposition are linked to cognitive decline in type 2 diabetes. However, whether and how subcortical atrophy is related to Alzheimer’s pathology in diabetes remains unclear. This study therefore aimed to investigate subcortical structural alterations induced by diabetes and the relationship between subcortical alteration, Alzheimer’s pathology and cognition.

**Methods:**

Participants were 150 patients with type 2 diabetes and 598 propensity score-matched controls without diabetes from the Alzheimer’s Disease Neuroimaging Initiative. All subjects underwent cognitive assessments, magnetic resonance imaging (MRI), and apolipoprotein E (ApoE) genotyping, with a subset that underwent amyloid positron emission tomography (PET) and cerebrospinal fluid (CSF) assays to determine cerebral β-amyloid deposition (*n* = 337) and CSF p-tau (*n* = 433). Subcortical structures were clustered into five modules based on Pearson’s correlation coefficients of volumes across all subjects: the ventricular system, the corpus callosum, the limbic system, the diencephalon, and the striatum. Using structural equation modeling (SEM), we investigated the relationships among type 2 diabetes, subcortical structural alterations, and AD pathology.

**Results:**

Compared with the controls, the diabetic patients had significant reductions in the diencephalon and limbic system volumes; moreover, patients with longer disease duration (>6 years) had more severe volume deficit in the diencephalon. SEM suggested that type 2 diabetes, age, and the ApoE ε4 allele (ApoE-ε4) can affect cognition *via* reduced subcortical structure volumes (total effect: age > ApoE-ε4 > type 2 diabetes). Among them, age and ApoE-ε4 strongly contributed to AD pathology, while type 2 diabetes neither directly nor indirectly affected AD biomarkers.

**Conclusion:**

Our study suggested the subcortical atrophy mediated the association of type 2 diabetes and cognitive decline. Although both type 2 diabetes and AD are correlated with subcortical neurodegeneration, type 2 diabetes have no direct or indirect effect on the cerebral amyloid deposition and CSF p-tau.

## Introduction

With an increasing incidence and associated economic burden, type 2 diabetes continues to be a worldwide public healthcare concern. Compared with people without diabetes, patients with type 2 diabetes tend to have greater cognitive dysfunction in multiple domains (Biessels and Despa, 2018), especially in memory, processing speed, and executive function. Although epidemiological studies have widely reported the association between type 2 diabetes and Alzheimer’s disease (AD), the current understanding of the mechanism underlying this relationship remains rudimentary.

Subcortical structures play pivotal roles in the cognitive, emotional and social functions of humans (Johnson, 2005). Previous neuroimaging studies have demonstrated that subcortical atrophy and its associated altered synaptic plasticity are early signs of cognitive impairment both in diabetes and AD (Moran et al., 2013; Chen et al., 2017), and investigation of the association between cerebral alterations in type 2 diabetes and AD pathological biomarkers may offer an entry point for understanding the similarities and differences in the cognitive decline mechanisms of the two diseases (Kandimalla et al., 2017). However, it is well known that cognitive decline is a complex multifactorial process and could be affected either directly or indirectly by age, sex, the apolipoprotein E ε4 allele (ApoE-ε4), education and the clinical characteristics of type 2 diabetes (Moran et al., 2019). Few published studies have reported how subcortical atrophy is associated with the typical pathology of AD in type 2 diabetes, and it is also unclear whether subcortical structural changes mediate the relationship between diabetes and cognitive decline.

We hypothesized that the associations between subcortical structural alterations and AD pathology in type 2 diabetes patients can be disentangled by calculating the direct and indirect effects of type 2 diabetes, age and ApoE-ε4 on each process. Structural equation modeling (SEM) is widely performed in psychology, sociology, and economics, and allows us to evaluate networks of multiple known or theoretical casual relationships to understand complex systems (Cheung, 2019).

Therefore, in this study, we aimed to investigate the complex associations between type 2 diabetes-induced subcortical structural alterations and AD pathological biomarkers in an elderly patient sample using two separate methods. First, we evaluated major subcortical structural alterations in patients with type 2 diabetes, after conducting propensity score matching (PSM) to reduce the potential biases from age, sex, and ApoE-ε4. Second, we conducted SEM analysis to extract the direct and indirect effects of type 2 diabetes, age, and ApoE-ε4 on major subcortical structural alterations, AD pathological biomarkers and cognition.

## Materials and Methods

### Data Source

Data were obtained from the Alzheimer’s Disease Neuroimaging Initiative (ADNI) database (adni.loni.usc.edu). The ADNI was launched in 2003 as a multisite study that aims to investigate clinical, serial magnetic resonance imaging (MRI), positron emission tomography (PET), genetic, and biological markers for the early detection and tracking of mild cognitive impairment (MCI) and early AD. The ADNI has recruited more than 2500 older participants (aged 55–90 years) with normal cognition, mild cognitive impairment, or AD. Exclusion criteria of the ADNI study include a Hachinski ischemic score > 4, current depression, a history of psychiatric or neurologic disorders, alcohol, or substance dependence in the last 2 years, less than 6 years of education, and lack of fluency in English or Spanish. Further information can be found on the official website and in their publications. The authors of this paper were granted access to the ADNI database, and submission of our paper was permitted by the ADNI Data Sharing and Publications Committee.

### Participant Selection

**FIGURE 1:**
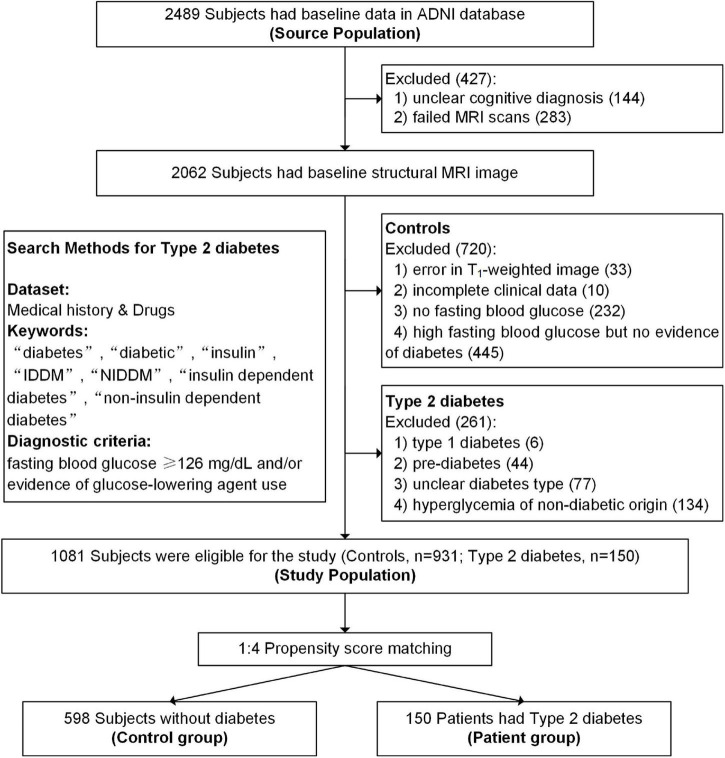
Flowchart of the study population.

Type 2 diabetes was identified using the guidelines recommended by the American Diabetes Association (American Diabetes Association [ADA], 2019) and included people who had a fasting blood glucose ≥ 126 mg/dL and/or evidence of type 2 diabetes medication use according to their medical history and medication records. Participants with incomplete clinical information, unclear cognitive diagnosis, failed MRI scans, poor image quality, and unclear diabetes type were excluded. Detailed search methods and flowcharts can see in Figure 1. All participants provided informed consent at their first visit, and full details of ethics approval are available on the ADNI website.

### Clinical, Genetic, and Cognitive Information

Demographic information, baseline cognitive status (AD, MCI, or normal cognition), ApoE-ε4 carrier status, vascular risk factors, glucose metabolism characteristics, and cognitive performance are shown in Table 1. ApoE-ε4 positivity was defined as carrying one or more ε4 alleles. Vascular risk factors were evaluated according to physical examinations and medical records. Hypertension, stroke and dyslipidemia were determined by diagnosis and treatment records in the medical history.

**TABLE 1:** Clinical participant characteristics.

Characteristic	Type 2 diabetes (*n* = 150)	Controls (*n* = 598)	*p-*value
**Demographics**			
Age, years	73.34 ± 6.99	73.38 ± 7.14	0.949
Sex (male)	106 (70.7)	424 (70.9)	0.955
Education, years	15.59 ± 2.97	15.80 ± 2.80	0.415
APOE-ε4 (ε4 positive)	61 (40.7)	267 (44.6)	0.379
GDS score	1.53 ± 1.49	1.29 ± 1.37	0.072
**Glucose metabolism**			
Fasting glucose, mg/dl	129.06 ± 42.92	94.85 ± 10.60	<0.001
Median duration (years), (IQR)	6 (4–11)	–	
**Medication use**			
Oral hypoglycemic agents	111 (74)	–	
Insulin use	16 (10.7)	–	
Insulin with oral agents	5 (3.3)	–	
**Vascular risk factors**			
Stroke history	4 (2.7)	4 (0.7)	0.056
Weight, kg	84.16 ± 17.14	78.75 ± 14.28	<0.001
BMI, kg/m^2^	28.65 ± 4.88	26.94 ± 4.36	<0.001
Dyslipidemia	66 (44.0)	150 (25.1)	<0.001
Hypertension	95 (63.3)	244 (40.8)	<0.001
Systolic BP, mmHg	137.08 ± 16.84	135.32 ± 16.90	0.254
Diastolic BP, mmHg	73.95 ± 9.61	75.10 ± 9.40	0.185
**Cognition**			
Cognitive status (%), NC/MCI/AD	32.0/51.3/16.7	32.1/53.5/14.4	0.767
MMSE-all sample	27.41 ± 2.48	27.47 ± 2.60	0.821
MMSE-NC	28.90 ± 1.08	29.11 ± 1.02	0.201
MMSE-MCI	27.74 ± 1.78	27.66 ± 1.90	0.725
MMSE-AD	23.56 ± 2.40	23.09 ± 2.47	0.405
Memory*	0.03 ± 1.06	–0.01 ± 0.99	0.656
Verbal fluency*	–0.02 ± 1.01	0.06 ± 1.00	0.753
Attention*	–0.09 ± 0.81	0.02 ± 0.85	0.334
Executive function (time)*	0.00 ± 0.88	–0.03 ± 0.88	0.757
**AD biomarkers**			
Amyloid PET, SUVR^†^	1.22 ± 0.22	1.19 ± 0.22	0.275
CSF p-tau^‡^	26.98 ± 13.76	27.91 ± 15.52	0.597
CSF t-tau^‡^	279.17 ± 121.32	291.47 ± 135.45	0.426
CSF Aβ1–42^‡^	850.19 ± 362.52	912.36 ± 353.10	0.211
CSF p-tau/Aβ1–42^‡^	0.04 ± 0.03	0.04 ± 0.03	0.600
CSF t-tau/Aβ1–42^‡^	0.41 ± 0.30	0.39 ± 0.27	0.555

*Data are presented as the means ± standard deviation and n (%) unless otherwise indicated. MMSE, Mini-Mental State Examination; CDR-SB, Clinical Dementia Rating Sum of Boxes; ADAS-cog, Alzheimer’s Disease Assessment Scale-cognitive subscale; BMI, body mass index; BP, blood pressure; CSF, cerebrospinal fluid.*

*Standardized Z-transformation. ^†^Amyloid PET data were available for n = 337 participants. ^‡^CSF samples were available for n = 433 participants.

Details of the neuropsychological assessment procedures are provided in ADNI’s previous publications. The following assessments were used in our study: Geriatric Depression Scale (depression scores), Mini-Mental State Examination (global cognition), Alzheimer’s Disease Assessment Scale–cognitive subscale (global cognition), Rey Auditory Verbal Learning Test (memory), category fluency (verbal fluency), Boston Naming Test (verbal fluency), Digit Span Test forward and backward (attention), and Trail-Making Test A and B (motor speed and executive function).

### Propensity Score Matching

We performed PSM to reduce the potential confounding bias arising from non-comparable demographic characteristics of type 2 diabetes patients and controls. The covariates considered in the propensity score calculation included age, sex, education, ApoE-ε4 carrier status, and cognitive status; the included variables were expected to influence both cerebral volume and cognitive function. The propensity scores were estimated using SPSS (SPSS Statistics 22.0; IBM, Chicago, IL). Type 2 diabetes patients and control subjects were then matched 1:4 using the nearest neighbor matching method based on their corresponding propensity scores (Rassen et al., 2012). Each patient was randomly matched to the four normal control subjects with the closest propensity score (Austin, 2011), that is, within –0.1 to +0.1.

### Alzheimer’s Disease Pathology

Acquisition and processing details of the 18F florbetapir amyloid (AV45)-PET scans in the ADNI study have been reported previously (Jagust et al., 2015). AV45-PET was computed for the regionally standardized uptake value ratios (SUVRs) relative to the whole cerebellum for 7 regions of interest (ROIs), including the temporal, anterior cingulate, orbital frontal, posterior cingulate, and parietal cortices and the precuneus. Sample collection and processing protocols of baseline cerebrospinal fluid (CSF) biomarkers have also been described in previous publications (Shaw et al., 2009). An Innogenetics immunoassay kit (INNO-BIA AlzBio3; Ghent, Belgium) was used to detect β-amyloid 1–42 (Aβ1–42), phosphorylated tau (p-tau) and total tau (t-tau).

### Image Processing and Quality Control

Details on the acquisition protocol for the MR images were reported previously (Jack et al., 2008). We used FreeSurfer v6.0 to perform automated segmentation and volumetric measurement of subcortical structures from T1-weighted images. After excluding areas with incomplete scan coverage such as the brainstem and cerebellum, 27 subcortical structures and their volumes were obtained for further analyses. The segmentation results were individually examined by an expert radiologist. Then, we checked study-wide statistics to detect non-normally distributed data and major outliers. A statistical outlier was defined as >3 standard deviations away from the mean. The agreement of volume measurements between the ADNI and us estimated by the intraclass correlation coefficient for main subcortical structure was excellent (>0.8) and ranged between 0.890 and 0.972. The total intracranial volume (ICV) of each subject was collected for head size correction. We performed a regression-based covariance method to adjust the observed volumes by an amount proportional to the difference between an individual’s observed ICV and the mean ICV for all participants (Voevodskaya et al., 2014), as below.


$$R\,O\,I\,\left(a\,d\,j\,u\,s\,t\,e\,d\right)=R\,O\,I\,\left(o\,b\,s\,e\,r\,v\,e\,d\right)-B\,\left(I\,C\,V_{i}-I\,C\,V_{m\,e\,a\,n}\right)$$

*ICVi* is the ICV of the subject, *ICVmean* is the average ICV across all subjects, and *B* is the slope of the regression line between each ROI and *ICVi*.

### Clustering of Volumetric Traits

Pearson’s correlation coefficients were calculated between subcortical structure volumes across all participants. Next, hierarchical clustering was performed on the correlation matrix using the pheatmap package in R software with the “complete distance” method. All structures were clustered into five modules. The volume of each module was the sum of the volumes of the ROIs within that module.

### Statistical Analyses

We used SPSS for basic statistical analyses. Clinical characteristics were compared using the independent-samples *t*-test (or Mann–Whitney *U*-test) for continuous variables and the chi-square test (or Fisher’s exact test) for categorical variables. ANCOVA was used to test for group differences in subcortical structural volumes. Age, sex, ApoE-ε4 carrier status, hypertension, dyslipidemia, weight, depression scores, stroke history and BMI were always considered covariates in all analyses. Cohen’s *d* and the 95% confidence interval were calculated to evaluate the effect size. To further investigate the characteristics of regional brain changes in type 2 diabetes, we conducted stratified analyses to explore whether the duration of diabetes (≤ 6 years or > 6 years) may be related to subcortical structural volumes; we chose a cut-off of 6 years, as this was the median duration of illness for type 2 diabetes patients in the current sample. In this study, we reported uncorrected *P*-values with the significance threshold determined by false discovery rate (FDR) correction within each module.

A two-step analysis approach (measurement model and structural model) for the SEM was conducted to estimate the associations between subcortical structures and AD biomarkers and cognitive function, using Mplus v8.0 and the R Lavaan package. Confirmatory factor analysis (CFA) was used to combine multiple cognitive assessments to a single latent variable, cognitive function. Convergent validity was assessed using standard factor loadings ≥ 0.6, average variance extracted (AVE) ≥ 0.5, and composite reliability (CR) ≥ 0.7 (Fornell and Larcker, 1981). After implementation of the measurement model, we constructed the structural model. Model fitness was evaluated by the following fit indices: incremental fit index (IFI), comparative fit index (CFI), Tucker-Lewis index (TLI), standardized root mean residual (SRMR), and root mean square error of approximation (RMSEA). IFI ≥ 0.95, CFI ≥ 0.95, TLI ≥ 0.95, SRMR ≤ 0.08, and RMSEA ≤ 0.06 were considered a “good” model fit (Kline, 2011).

## Results

### General Participant Characteristics

After PSM, 748 matched participants were available (detail information is shown in Supplementary Table 2). In this matched cohort, the type 2 diabetes group and control group had no significant differences in terms of demographic characteristics, performance in the four major cognitive domains, depression scores or stroke history (all *p* > 0.05), as shown in Table 1. As expected, patients with type 2 diabetes had a higher BMI (*p* < 0.001), weight (*p* < 0.001) and fasting glucose (*p* < 0.001) than the controls. Moreover, those with type 2 diabetes had a higher risk of hypertension (*p* < 0.001) and dyslipidemia (*p* < 0.001). A total of 111 patients with type 2 diabetes used oral hypoglycemic agents and 16 used insulin treatment (five of whom also used oral agents). The details of the diabetes medications are listed in Supplementary Table 1.

### Alzheimer’s Disease Biomarkers

AV45-PET scans were available for 337 participants (type 2 diabetes: *n* = 64; controls: *n* = 273), and we did not find a statistically significant difference in cerebral amyloid between the two groups (*p* = 0.275). CSF samples were available for 57.8% of the total cohort (type 2 diabetes: *n* = 79; controls: *n* = 354). There were no group differences in CSF Aβ1–42 (*p* = 0.211), CSF t-tau (*p* = 0.426), CSF p-tau (*p* = 0.597), t-tau/ Aβ1–42 (*p* = 0.555), or p-tau/ Aβ1–42 (*p* = 0.60) (Table 1).

### Module Clustering of Subcortical Structures

**FIGURE 2:**
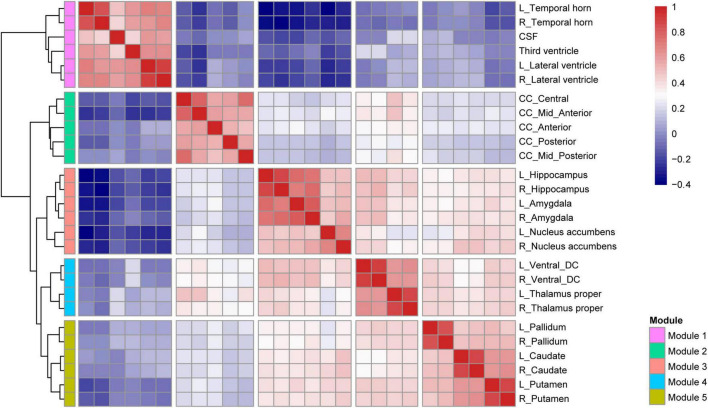
Hierarchical clustering and correlation heatmap of 27 subcortical structures across all subjects.

As shown in Figure 2, 27 subcortical structures were clustered into five modules based on the Pearson’s correlation coefficient matrix of subcortical structural volumes across all subjects. Module 1 included the CSF, lateral ventricle, third ventricle, and temporal horn of the lateral ventricle. Module 2 included five structures belonging to the corpus callosum. Module 3 comprised three structures, including the hippocampus, amygdala, and nucleus accumbens. Module 4 includes the thalamus and ventral diencephalon (containing the hypothalamus, red nuclei, basal forebrain, ventral tegmentum, and geniculate nuclei). Module 5 comprised three structures, including the bilateral caudate, putamen, and pallidum.

### Group Differences in Subcortical Structural Volumes

**FIGURE 3:**
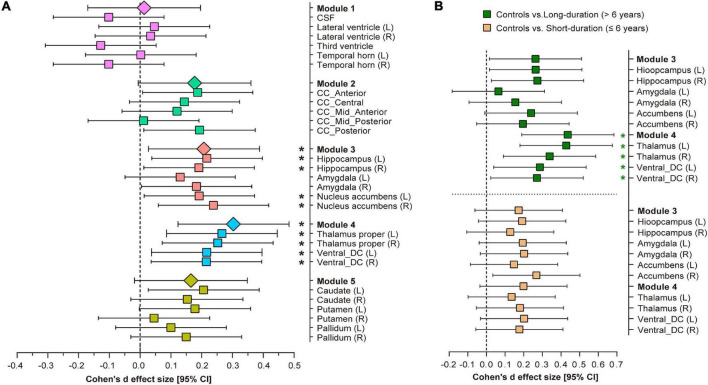
Volumetric trait module-based analysis of subcortical structures between type 2 diabetes patients and controls. **(A)** Cohen’s *d* effect size and 95% confidence interval (CI) for the differences in subcortical structures between controls and type 2 diabetes patients. **(B)** Cohen’s *d* effect size and 95% CI for the subcortical volume differences in Modules 3 and 4 between long-duration (green pattern) and short-duration (orange pattern) diabetes patients, vs. controls. Cohen’s *d* effect size and 95% CI were corrected for age, sex, ApoE-ε4 carrier status, hypertension, dyslipidemia, weight, BMI, depression scores and stroke history. *Indicates *p* < 0.05 after false discovery rate (FDR) correction per module. L, left hemisphere; R, right hemisphere; CSF, cerebrospinal fluid; CC, corpus callosum; DC, diencephalon.

Comparisons of the subcortical structure volumes between the controls and type 2 diabetes patients are shown in Figure 3A and Supplementary Table 3. Compared with the controls, patients with type 2 diabetes showed decreased volumes in Module 3 (*p* = 0.018, partial η^2^ = 0.008) and Module 4 (*p* = 0.001, partial η^2^ = 0.015), including the bilateral thalamus (left: *p* = 0.004; right: *p* = 0.007), bilateral ventral diencephalon (left: *p* = 0.013; right: *p* = 0.015), left hippocampus (*p* = 0.020), and right nucleus accumbens (*p* = 0.010). Additionally, we also found reduced volumes of the left caudate, right hippocampus, left nucleus accumbens, anterior corpus callosum, and posterior corpus callosum in patients with type 2 diabetes, but none of these results survived correction for multiple comparisons.

### Influences of Diabetes Duration on Subcortical Structural Volumes

After splitting the type 2 diabetes patients into short-duration (≤6 years) and long-duration (> 6 years) subgroups, we found a significant volumetric difference only in Module 4 among the three groups (*p* = 0.001, partial η^2^ = 0.018, Supplementary Table 4). *Post-hoc* analyses indicated that long-duration patients exhibited a lower thalamic volume (left: *p* < 0.001, Cohen’s *d* = 0.406; right: *p* = 0.009, Cohen’s *d* = 0.323) and ventral diencephalon volume (left: *p* = 0.025, Cohen’s *d* = 0.279; right: *p* = 0.03, Cohen’s *d* = 0.265) than controls, but we did not detect any significant volumetric differences between short-duration patients and controls, or between the two subgroups of diabetes patients (Figure 3B). The complete results of the between-group analysis are listed in Supplementary Tables 4, 5.

### Structural Equation Modeling

CFA generated a single factor to represent overall cognitive function according to the following assessments: Rey Auditory Verbal Learning Test (immediate recall), Boston Naming Test, Trail-Making Test A and B, and category fluency. The model fit the data well; AVE (0.589) was greater than 0.5, CR (0.839) was greater than 0.7, and all standardized factor loadings were above 0.6.

**FIGURE 4:**
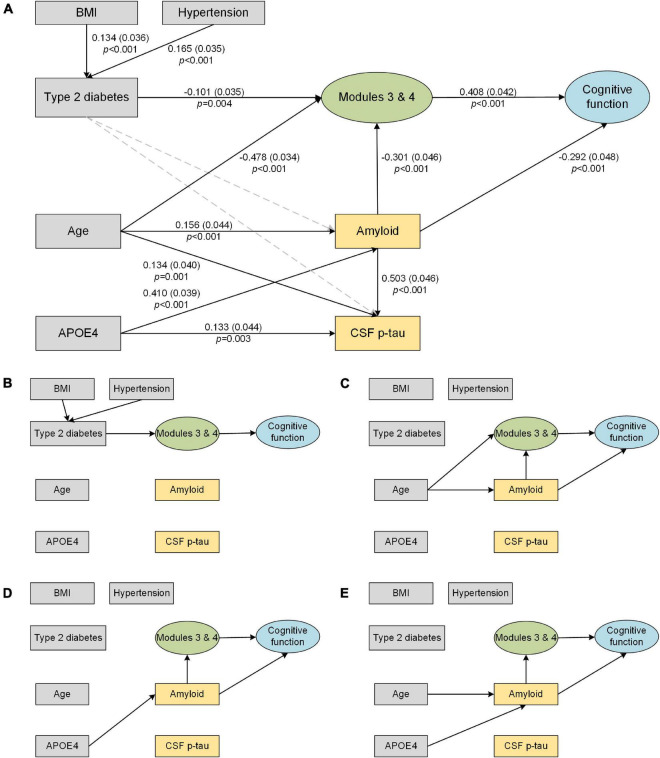
Structural equation model of subcortical structures (Modules 3 and 4), AD biomarkers and cognitive function. Solid line arrows are statistically significant associations in the model. The direct and indirect effects observed in the figure are summarized in Table 2. **(A)** Final structural equation model. The standardized coefficients, standard errors (in brackets), and *P*-values are shown beside the solid arrows. **(B–E)** Direct and indirect pathways from individual factors to cognition. **(B)** Path from type 2 diabetes to cognition. **(C)** Path from age to cognition. **(D)** Path from APOE4 to cognition. **(E)** Path from AD biomarkers to cognition. BMI, body mass index; CSF, cerebrospinal fluid; p-tau, phosphorylated tau.

The results of the significant associations in the SEM analysis are shown in Figure 4A. The final model fits the data well according to these indices: χ^2^*/df* (1.574) was less than 3; TLI (0.950), CFI (0.955), and IFI (0.956) were all greater than 0.95; and SRMR (0.051) and RMSEA (0.046) were both less than 0.06. The direct and indirect effects observed in Figure 4A are summarized in Table 2. Here, we found that (1) hypertension (coefficient = 0.165) and higher BMI (coefficient = 0.134) directly contributed to type 2 diabetes; (2) age (coefficient = –0.478) and type 2 diabetes (coefficient = –0.101) have significant direct effects on the subcortical structural volume; in addition to ApoE-ε4, these three factors can affect cognition *via* the subcortical structural volume, with the effect of age pathway (coefficient = –0.214) having a biggest effect than the type 2 diabetes (coefficient = –0.041) and ApoE-ε4 pathways (coefficient = –0.050); (3) age and ApoE-ε4 also affected cognition indirectly through cerebral amyloid deposition (age pathway: coefficient = –0.046; ApoE-ε4 pathway: coefficient = –0.120), while type 2 diabetes was neither directly nor indirectly associated with either cerebral amyloid deposition (*p* = 0.618) or increased CSF p-tau (*p* = 0.519); (4) cerebral amyloid deposition can affect cognition directly (coefficient = –0.362) or indirectly through subcortical structures (coefficient = –0.044), but, CSF p-tau was not associated with either subcortical structural volume or cognition.

**TABLE 2:** Direct and indirect effects seen in the final SEM.

Effect	Path	Estimate (std. error)	*p-*value
Total	BMI to type 2 diabetes	0.134 (0.036)	<0.001
Direct	BMI to type 2 diabetes	0.134 (0.036)	<0.001
Total	HTN to type 2 diabetes	0.165 (0.035)	<0.001
Direct	HTN to type 2 diabetes	0.165 (0.035)	<0.001
Total	BMI to subcortical structures	–0.014 (0.006)	0.023
Indirect	BMI to type 2 diabetes to subcortical structures	–0.014 (0.006)	0.023
Total	HTN to subcortical structures	–0.017 (0.007)	0.015
Indirect	HTN to type 2 diabetes to subcortical structures	–0.017 (0.007)	0.015
Total	Type 2 diabetes to subcortical structures	–0.101 (0.035)	0.004
Direct	Type 2 diabetes to Subcortical structures	–0.101 (0.035)	0.004
Total	Age to subcortical structures	–0.525 (0.032)	<0.001
Direct	Age to subcortical structures	–0.478 (0.034)	<0.001
Indirect	Age to amyloid to subcortical structures	–0.047 (0.015)	0.002
Total	ApoE-ε4 to subcortical structures	–0.123 (0.023)	<0.001
Indirect	ApoE-ε4 to amyloid to subcortical structures	–0.123 (0.023)	<0.001
Total	Age to amyloid	0.156 (0.044)	<0.001
Direct	Age to amyloid	0.156 (0.044)	<0.001
Total	ApoE-ε4 to amyloid	0.410 (0.039)	<0.001
Direct	ApoE-ε4 to amyloid	0.410 (0.039)	<0.001
Total	Age to CSF p-tau	0.213 (0.041)	<0.001
Direct	Age to CSF p-tau	0.134 (0.040)	0.001
Indirect	Age to amyloid to CSF p-tau	0.079 (0.023)	<0.001
Total	ApoE-ε4 to CSF p-tau	0.339 (0.040)	<0.001
Direct	ApoE-ε4 to CSF p-tau	0.133 (0.044)	0.003
Indirect	ApoE-ε4 to amyloid to CSF p-tau	0.206 (0.044)	0.003
Total	Amyloid to CSF p-tau	0.503 (0.046)	<0.001
Direct	Amyloid to CSF p-tau	0.503 (0.046)	<0.001
Total	Type 2 diabetes to cognition	–0.041 (0.015)	0.006
Indirect	Type 2 diabetes to subcortical structures to cognition	–0.041 (0.015)	0.006
Total	Age to cognition	–0.260 (0.027)	<0.001
Indirect	Age to subcortical structures to cognition	–0.195 (0.025)	<0.001
Indirect	Age to amyloid to cognition	–0.046 (0.015)	0.002
Indirect	Age to Amyloid to subcortical structures to cognition	–0.019 (0.006)	0.003
Total	ApoE-ε4 to cognition	–0.170 (0.024)	<0.001
Indirect	ApoE-ε4 to amyloid to cognition	–0.120 (0.023)	<0.001
Indirect	ApoE-ε4 to amyloid to subcortical structures to cognition	–0.050 (0.011)	0.001
Total	Subcortical structures to cognition	0.408 (0.042)	<0.001
Direct	Subcortical structures to cognition	0.408 (0.042)	<0.001
Total	Amyloid to cognition	–0.415 (0.043)	<0.001
Direct	Amyloid to cognition	–0.292 (0.048)	<0.001
Indirect	Amyloid to subcortical structures to cognition	–0.123 (0.022)	<0.001

*BMI, body mass index; HTN, hypertension; ApoE, apolipoprotein E; CSF, cerebrospinal fluid.*

## Discussion

In this work, we identified subcortical structural alterations in type 2 diabetes and its potential effect on AD pathological biomarkers and cognition. Our main findings were that (1) compared with those of controls, the volumes of the limbic system and diencephalon were significantly reduced in type 2 diabetes patients, particularly in long-duration (>6 years) diabetes patients; (2) type 2 diabetes had no significant direct or indirect effect on AD biomarkers (cerebral amyloid and CSF p-tau), but did have a significant indirect effect on cognitive function through subcortical structural atrophy; and (3) the effect of subcortical structural volume on cognition was driven by type 2 diabetes, age and APOE4, but the effect of type 2 diabetes was smaller than that of the other two factors.

Prior studies have revealed several modular organizations both among cortical and subcortical structures (Chen et al., 2008; Yuan et al., 2019), and our modular clustering results of subcortical structures are essentially consistent with the previous ones (Yuan et al., 2019). Some structures have similar patterns of volumetric alterations although they are in distinct spatial locations, they may follow a certain pattern of change in a disease state. Our major finding of decreased volumes in Module 3 (limbic system) and Module 4 (diencephalon) in type 2 diabetes is generally in line with previous neuroimaging studies (Moran et al., 2013; Chen et al., 2017). The limbic system supports a variety of functions including emotion, behavior, motivation, long-term memory, and olfaction (Morgane et al., 2005). The thalamus serves as a pivotal relay station to the cerebral cortex, which plays an important role in multiple cognitive processes (Huda et al., 2019). The diencephalon acts as a primary relay to the cortex and as a processing center for sensory information and autonomic control (Versteeg et al., 2017) and plays an important role in memory and emotion *via* connections to the limbic system.

Brain atrophy in type 2 diabetes is often linked to “cerebral insulin resistance.” Insulin receptors are abundantly expressed throughout the brain on neurons and glial cells, especially in the hypothalamus, hippocampus, striatum, cerebellum, and cortical regions (Kullmann et al., 2020). Insulin binds to its receptors in the brain and modulates glucose and energy metabolism, and it is indirectly involved in synaptic function and neurotransmitter activity (Kleinridders et al., 2014). Disruption of insulin action in the brain leads to alterations in not only metabolic disorders but also neurodegenerative diseases (Heni et al., 2015). It has been reported that cerebral insulin resistance in type 2 diabetes is associated with an altered neural response to insulin and disorders of food cravings and hunger, which may induce cognitive dysfunction by affecting dopamine signaling (Kleinridders et al., 2015), impairing the blood-brain barrier (Rhea and Banks, 2019), and damaging hippocampal synaptic plasticity (Biessels and Reagan, 2015).

Moreover, we observed more severe volume deficits in Module 4 (thalamus and ventral diencephalon) in patients with long-duration diabetes. Longitudinal studies have shown that cerebral atrophy progressively worsens with the course of diabetes beyond the levels expected for normal aging (van Elderen et al., 2010). Our results may reflect a greater chance of exposure to the hyperglycemic environment in patients with longer illness durations. Unfortunately, detailed information regarding glycemic control, such as glycated hemoglobin, was not available in the ADNI database, and we cannot quantify the effect of the severity of diabetes on cerebral structures. Simply calculating disease burden as diabetes duration is unsatisfactory, given the systemic nature of the damage caused by diabetes, and so this finding can be viewed only as a conservative conclusion for similar studies.

Multiple studies have reported that well-controlled diabetes is associated with a lower risk of dementia, and treatment status may alter the relationship between type 2 diabetes and the AD biomarker profile (McIntosh et al., 2019). Typical diabetes medications, such as metformin, can decrease p-tau and β-amyloid in both diabetes and AD animal models (Li et al., 2012; Chen et al., 2021). To explain the benefit of glucose management as precisely as possible, we also attempted to examine whether anti-diabetic medication treatment can regulate AD pathology and subcortical structures in our samples. When we classified patients as treated and untreated according to their anti-diabetic medication records, we found that the treated group had greater CSF Aβ1-42 than the untreated group (*F* = 4.792, *P* = 0.033), suggesting that diabetic medication treatment may help cerebral amyloid clearance. We observed lower subcortical structural volume in the treated group than the untreated group, but this trend did not rise to statistical significance. It could be that the diabetic duration of the two groups was indeed different (average duration of the untreated group: 6.8 years; average duration of treated group: 9.6 years). Because we have reported that long-duration patients had more severe volume loss. Furthermore, when we classified anti-diabetic medication into insulin-sensitizing drugs (metformin and thiazolidinedione) and other drugs, we did not find any group differences in either AD pathology or subcortical structures.

The major concern of this study was the relationship between type 2 diabetes and AD biomarkers. SEM allowed us to simultaneously observe the direct and indirect effects for each variable and explore whether type 2 diabetes is independently associated with the effect of other cognitive risk factors, which is impossible in simple group comparisons. Additionally, the comparability of the coefficients on the arrow going to a particular outcome also allowed us to quantify the effects of each factor on subcortical atrophy and cognition directly, another strength of the SEM approach.

As expected, type 2 diabetes, age and ApoE-ε4 were significant predictors of subcortical structural atrophy and cognitive decline (Figures 4B–D), which is consistent with known risk factors for dementia (Livingston et al., 2017). Our results suggest that the effect of type 2 diabetes on cognition is much smaller than that of age and ApoE-ε4 (coefficient: age > ApoE-ε4 > type 2 diabetes; Table 2). It is well known that cognitive abilities rise from infancy to young adulthood and then are either maintained or decline into old age (Spreng and Turner, 2019). Aging leads to multi-system dysfunction at the cellular or tissue level and contributes to multiple brain changes and cognitive decline (Fjell et al., 2014). The ApoE-ε4 variant was reported to be the largest known non-modifiable risk factor for typical late-onset, sporadic AD (Livingston et al., 2017). Therefore, to reduce potential bias, it is necessary to take age and ApoE-ε4 into account when analyzing the influence of type 2 diabetes on cognition in future research.

Furthermore, we also examined the effect of type 2 diabetes on AD pathology. In the current study, although the type 2 diabetes and AD pathways work together in inducing the atrophy of subcortical structures and promoting cognitive decline, we did not find associations between type 2 diabetes and brain amyloid or CSF p-tau levels (Figure 4E). Observations from both post-mortem neuropathological studies (Dos Santos Matioli et al., 2017) and prospective population-based cohort studies (Moran et al., 2015) have also indicated that type 2 diabetes does not influence β-amyloid deposition. It has been also reported that type 2 diabetes may promote neurodegeneration through its effects on other non-Alzheimer pathophysiology. For example, the non-enzymatic glycation of proteins by D-ribose may result in irreversible production of advanced glycation end products (Lu et al., 2021), subsequently further activating the inflammatory response and potentiating neurodegeneration and cognitive impairments (Soboleva et al., 2019). Microvascular endothelial cell injury (Yin et al., 2021) and mitochondrial dysfunction (Potenza et al., 2021) due to microvascular complications and insulin-signaling dysregulation also lead to increased oxidative stress. In addition, blood-brain barrier dysfunction (Takechi et al., 2017) and abnormal metabolomics (Song et al., 2017) also precede cognitive decline and neurodegeneration in diabetes. These above mechanisms might help explain the paradox wherein type 2 diabetes is always correlated with an increased risk of AD, but seemingly not with increased amyloid deposition.

Our study has some limitations. It was reported that anti-diabetic medications can reduce the incidence of cognitive impairment in type 2 diabetes (Cukierman-Yaffe et al., 2020; Samaras et al., 2020). Unfortunately, as the primary objective of the ADNI cohort did not focus on diabetes, we lacked information on glycosylated hemoglobin, insulin levels and treatment compliance, which are important references for judging the disease conditions and treatment regimens of patients with type 2 diabetes, and can also provide extra clues for exploring whether the insulin resistance had improved and whether standardized management could slow subcortical atrophy and pathological deposition or improve cognition. In addition, potential recruitment bias may have limited the generalizability of our results, as subjects who participated in the ADNI cohort were screened for many cerebrovascular disorders and thus had fewer vascular risk factors than the general population. On the other hand, this could be a strength of our study, as we can better examine how type 2 diabetes itself is related to AD pathology under a less severe vascular burden. Third, different neuropathological subtypes of AD may have subtle differences in brain alterations (Rabinovici et al., 2010; Jagust, 2018), whereas we were unable to distinguish early onset AD or late-onset AD in the ADNI study, which may also influence the results.

Fourth, we restricted our current analyses to subcortical structures and did not include cortical measurements, so it is difficult to conclude whether the cortical abnormalities caused by type 2 diabetes were also independent of AD pathology. Our current study is only the first step; next, we will explore the associations between cortical alterations and AD pathology in type 2 diabetes. Finally, given the complete and continued follow-up plan of the ADNI cohort, further longitudinal analyses are needed to validate whether the causality in the current results changes with aging.

In summary, our study suggests that type 2 diabetes may contribute to cognitive impairment indirectly through decreased subcortical structural volume particularly in the limbic system and diencephalon. Furthermore, although type 2 diabetes and AD are co-related with neurodegeneration and can affect cognition *via* their effects on subcortical structural volume, type 2 diabetes may have no direct or indirect effect on the pathological biomarkers of AD.

## Data Availability Statement

Publicly available datasets were analyzed in this study. This data can be found here: adni.loni.usc.edu.

## Ethics Statement

The studies involving human participants were reviewed and approved by the Alzheimer’s Disease Neuroimaging Initiative (ADNI) and ADNI Data Sharing and Publications Committee. The patients/participants provided their written informed consent to participate in this study.

## Author Contributions

WZ contributed to the study design and statistical analyses and wrote the manuscript. JL and ZQ contributed to the imaging processing and reviewed the manuscript. WZ and YB contributed to the revision of the manuscript. XZ, HZ, and YB participated in discussions of the results and reviewed the manuscript. BZ supervised the entire study and reviewed the manuscript, was the guarantor of this work and, as such, had full access to all the data in the study and took responsibility for the integrity of the data and the accuracy of the data analysis. All authors contributed to the article and approved the submitted version.

## Conflict of Interest

The authors declare that the research was conducted in the absence of any commercial or financial relationships that could be construed as a potential conflict of interest.

## Publisher’s Note

All claims expressed in this article are solely those of the authors and do not necessarily represent those of their affiliated organizations, or those of the publisher, the editors and the reviewers. Any product that may be evaluated in this article, or claim that may be made by its manufacturer, is not guaranteed or endorsed by the publisher.
