# Supplementary material for: Effects of Subcortical Atrophy and Alzheimer’s Pathology on Cognition in Elderly Type 2 Diabetes: The Alzheimer’s Disease Neuroimaging Initiative Study
Source: Front Aging Neurosci. 2022 Jan 31;14:781938. doi: 10.3389/fnagi.2022.781938 (PMC8841716; doi:10.3389/fnagi.2022.781938)
Supplement: Supplementary file 1 [file Data_Sheet_1.docx]

Supplementary Material

# Supplementary Figures and Tables

## Supplementary Tables

## Supplementary Table 1. Medication data for patients with type 2 diabetes

| Anti-diabetic medication | no. (%) |
| --- | --- |
| Medicine treatment | 118 (78.67) |
| Diet-exercise therapy | 9 (6.00) |
| Insulin | 16 (10.67) |
| Metformin | 88 (58.67) |
| Thiazolidinedione | 16 (10.67) |
| Sulfonylurea | 47 (31.33) |
| Meglitinides | 3 (2.00) |
| Alpha-glucosidase inhibitors | 1 (0.67) |
| DPP-4i | 2 (1.33) |
| GLP-1a | 1 (0.67) |
| SGLT-2i | 1 (0.67) |

Data are presented as number (%). DPP-4i, dipeptidyl peptidase 4 inhibitors; GLP-1a, glucagon-like peptide receptor 1 agonist; SGLT2i, sodium-glucose co-transporter2 inhibitors.

## Supplementary Table 2. General characteristic of the study population before and after propensity score matching

| Characteristic | Before Propensity Score-Matching | | | After Propensity Score-Matching | | |
| --- | --- | --- | --- | --- | --- | --- |
|  | Type 2 diabetes (n=150) | Control subjects (n=931) | *p* value | Type 2 diabetes (n=150) | Control subjects (n=598) | *p* value |
| **Demographics** |  |  |  |  |  |  |
| Age, years | 73.34 (6.99) | 73.46 (7.82) | 0.458 | 73.34 ± 6.99 | 73.38 ± 7.14 | 0.949 |
| Sex (male), no. (%) | 106 (70.7) | 865 (53.86) | <0.001† | 106 (70.7) | 424 (70.9) | 0.955 |
| Education, years | 15.59 (2.97) | 15.96 (2.81) | 0.098 | 15.59 ± 2.97 | 15.80 ± 2.80 | 0.415 |
| APOE-ε4 (ε4 positive), no. (%) | 61 (40.7) | 736 (45.83) | 0.248 | 61 (40.7) | 267 (44.6) | 0.379 |
| **Cognitive assessments** |  |  |  |  |  |  |
| Cognitive status (%), NC/MCI/AD | 32.0/51.3/16.7 | 27.1/52.4/20.4 | 0.390 | 32.0/51.3/16.7 | 32.1/53.5/14.4 | 0.767 |
| MMSE | 27.37 (2.49) | 26.91 (3.00) | 0.016† | 27.41 ± 2.48 | 27.47 ± 2.60 | 0.821 |
| ADAS-cog | 10.90 (5.73) | 11.30 (6.79) | 0.380 | 10.79 ± 5.73 | 10.59 ± 5.69 | 0.690 |
| Memory* | 0.22 ± 1.03 | 0.00 ± 1.00 | 0.783 | 0.03 ± 1.06 | -0.01 ± 0.99 | 0.656 |
| Verbal fluency* | -0.01 ± 0.98 | 0.01 ± 1.00 | 0.912 | -0.02 ± 1.01 | 0.06 ± 1.00 | 0.753 |
| Attention* | -0.06 ± 0.96 | 0.01 ± 0.99 | 0.479 | -0.09 ± 0.81 | 0.02 ± 0.85 | 0.334 |
| Executive function (time)* | 0.03 ± 0.86 | 0.00 ± 0.88 | 0.640 | 0.00 ± 0.88 | -0.03 ± 0.88 | 0.757 |
| **Vascular risk factors** |  |  |  |  |  |  |
| Weight, kg | 84.16 ± 17.14 | 75.67 ± 14.24 | <0.001† | 84.16 ± 17.14 | 78.75 ± 14.28 | <0.001† |
| BMI, kg/m^2^ | 28.65 ± 4.88 | 26.46 ± 4.68 | <0.001† | 28.65 ± 4.88 | 26.94 ± 4.36 | <0.001† |
| Dyslipidemia | 66 (44.0) | 416 (25.76) | <0.001† | 66 (44.0) | 150 (25.1) | <0.001† |
| Hypertension | 95 (63.3) | 647 (40.01) | <0.001† | 95 (63.3) | 244 (40.8) | <0.001† |
| Systolic BP, mmHg | 137.08 ± 16.84 | 135.09 ± 16.75 | 0.108 | 137.08 ± 16.84 | 135.32 ± 16.90 | 0.254 |
| Diastolic BP, mmHg | 73.95 ± 9.61 | 74.97 ± 9.75 | 0.317 | 73.95 ± 9.61 | 75.10 ± 9.40 | 0.185 |

Data are presented as the means ± SD and n (%) unless otherwise indicated. MMSE, Mini-Mental State Examination; CDR-SB, Clinical Dementia Rating Sum of Boxes; ADAS-cog, Alzheimer’s Disease Assessment Scale-cognitive subscale; BMI, body mass index; BP, blood pressure; CSF, cerebrospinal fluid. *Standardized Z-transformation. †*P* < 0.05 was considered significant.

## Supplementary Table 3. Group differences for each subcortical structure between the controls and patients with Type 2 diabetes

| Region of Interest | N | Controls | N | T2DM | F | *P-value* | partial η^2^ | *Cohen's d* | 95% CI for *Cohen's d* |
| --- | --- | --- | --- | --- | --- | --- | --- | --- | --- |
| Module 1: Ventricular system | 555 | 44989.3 (16475.9) | 145 | 44118.8 (16562.7) | 0.067 | 0.795 | 0.000 | 0.024 | (-0.159, 0.207) |
| CSF | 584 | 1332.6 (301.7) | 149 | 1343.3 (281.7) | 0.874 | 0.350 | 0.001 | -0.088 | (-0.268, 0.092) |
| Lateral ventricle (L) | 590 | 21636.6 (9405.6) | 149 | 21048.3 (8874.7) | 0.398 | 0.528 | 0.000 | 0.057 | (-0.123, 0.237) |
| Lateral ventricle (R) | 590 | 19250.0 (7985.0) | 149 | 18763.3 (7776.2) | 0.237 | 0.627 | 0.000 | 0.043 | (-0.137, 0.223) |
| Third ventricle | 597 | 1873.9 (573.2) | 149 | 1931.7 (569.4) | 1.999 | 0.158 | 0.003 | -0.124 | (-0.304, 0.056) |
| Temporal horn of lateral ventricle (L) | 575 | 975.7 (578.4) | 149 | 945.8 (505.4) | 0.022 | 0.881 | 0.000 | 0.013 | (-0.167, 0.193) |
| Temporal horn of lateral ventricle (R) | 577 | 920.6 (553.2) | 148 | 940.8 (555.6) | 1.237 | 0.266 | 0.002 | -0.097 | (-0.277, 0.083) |
|  |  |  |  |  |  |  |  |  |  |
| Module 2: Corpus callosum | 571 | 3242.0 (431.4) | 144 | 3178.9 (411.7) | 3.629 | 0.057 | 0.005 | 0.179 | (-0.004, 0.362) |
| Anterior corpus callosum | 591 | 878.9 (142.6) | 146 | 859.9 (135.5) | 4.293 | 0.039 | 0.006 | 0.195 | (0.016, 0.374) |
| Central corpus callosum | 598 | 447.0 (106.9) | 150 | 432.7 (78.8) | 2.522 | 0.113 | 0.003 | 0.148 | (-0.031, 0.327) |
| Middle anterior corpus callosum | 593 | 431.0 (84.2) | 150 | 422.4 (91.1) | 1.906 | 0.168 | 0.003 | 0.124 | (-0.055, 0.303) |
| Middle posterior corpus callosum | 590 | 476.7 (110.7) | 148 | 474.4 (99.3) | 0.003 | 0.955 | 0.000 | 0.005 | (-0.175, 0.185) |
| Posterior corpus callosum | 585 | 1011.6 (158.9) | 148 | 983.8 (171.7) | 4.183 | 0.041 | 0.006 | 0.195 | (0.014, 0.376) |
|  |  |  |  |  |  |  |  |  |  |
| Module 3: Limbic system | 586 | 10731.3 (1551.6) | 148 | 10518.4 (1537.0) | 5.635 | 0.018* | 0.008 | 0.198 | (0.018, 0.379) |
| Hippocampus (L) | 597 | 3443.6 (517.0) | 150 | 3363.9 (518.5) | 5.473 | 0.020* | 0.007 | 0.199 | (0.020, 0.379) |
| Hippocampus (R) | 596 | 3568.4 (532.9) | 150 | 3499.3 (575.9) | 4.398 | 0.036 | 0.006 | 0.178 | (0.001, 0.360) |
| Amygdala (L) | 598 | 1327.3 (281.6) | 150 | 1314.5 (288.5) | 1.954 | 0.163 | 0.003 | 0.120 | (-0.059, 0.299) |
| Amygdala (R) | 596 | 1538.8 (290.7) | 149 | 1519.1 (267.4) | 3.847 | 0.050 | 0.005 | 0.170 | (-0.009, 0.350) |
| Nucleus accumbens (L) | 595 | 418.5 (95.1) | 149 | 402.3 (95.5) | 3.949 | 0.047 | 0.005 | 0.174 | (-0.006, 0.353) |
| Nucleus accumbens (R) | 593 | 445.3 (88.5) | 149 | 426.7 (84.0) | 6.707 | 0.010* | 0.009 | 0.230 | (0.050, 0.409) |
|  |  |  |  |  |  |  |  |  |  |
| Module 4: Diencephalon | 583 | 20154.4 (1466.9) | 150 | 19707.0 (1471.0) | 11.342 | 0.001* | 0.015 | 0.291 | (0.111, 0.472) |
| Thalamus proper (L) | 595 | 6410.0 (577.1) | 150 | 6253.0 (575.4) | 8.572 | 0.004* | 0.012 | 0.259 | (0.079, 0.439) |
| Thalamus proper (R) | 595 | 6268.4 (562.7) | 150 | 6117.1 (525.7) | 7.234 | 0.007* | 0.010 | 0.240 | (0.060, 0.420) |
| Ventral diencephalon (L) | 591 | 3767.7 (351.9) | 150 | 3705.6 (366.2) | 6.246 | 0.013* | 0.009 | 0.224 | (0.045, 0.404) |
| Ventral diencephalon (R) | 591 | 3716.8 (339.3) | 150 | 3627.6 (343.6) | 5.921 | 0.015* | 0.008 | 0.216 | (0.037, 0.396) |
|  |  |  |  |  |  |  |  |  |  |
| Module 5: Striatum | 568 | 18859.2 (1723.8) | 144 | 18622.0 (1775.1) | 2.456 | 0.117 | 0.004 | 0.148 | (-0.035, 0.331) |
| Caudate (L) | 588 | 3290.8 (421.3) | 148 | 3214.4 (384.7) | 4.368 | 0.037 | 0.006 | 0.201 | (0.021, 0.382) |
| Caudate (R) | 588 | 3403.1 (438.4) | 147 | 3340.1 (435.0) | 5.599 | 0.107 | 0.004 | 0.155 | (-0.028, 0.336) |
| Putamen (L) | 589 | 4229.3 (528.6) | 148 | 4156.9 (540.4) | 2.660 | 0.103 | 0.004 | 0.149 | (-0.032, 0.330) |
| Putamen (R) | 592 | 4268.8 (550.0) | 146 | 4254.0 (563.7) | 0.141 | 0.708 | 0.000 | 0.035 | (-0.146, 0.216) |
| Pallidum (L) | 595 | 1883.4 (231.5) | 149 | 1869.8 (234.8) | 0.849 | 0.357 | 0.001 | 0.086 | (-0.094, 0.266) |
| Pallidum (R) | 594 | 1840.0 (215.0) | 148 | 1815.0 (231.2) | 2.449 | 0.118 | 0.003 | 0.149 | (-0.031, 0.329) |

ANCOVA, adjusted for age, sex, ApoE-ε4 carrier status, hypertension, dyslipidemia, weight, BMI, depression scores and stroke history. L, left hemisphere; R, right hemisphere; N, number of participants; CSF, cerebrospinal fluid; CI, confidence intervals. **p* value was considered significant after FDR correction within each module.

**Supplementary Table 4.** Influences of diabetes duration on the subcortical structural volume

| Region of Interest | N | Controls | N | Short-duration (≤6 years) | N | Long-duration (>6 years) | F | *P value* | partial η^2^ |
| --- | --- | --- | --- | --- | --- | --- | --- | --- | --- |
| Module 1: Ventricular system | 555 | 44989.3 (16475.9) | 76 | 43179.9 (15481.7) | 69 | 45152.9 (17733.9) | 0.295 | 0.745 | 0.000 |
| CSF | 584 | 1332.6 (301.7) | 79 | 1339.8 (272.6) | 70 | 1347.2 (293.5) | 0.441 | 0.644 | 0.001 |
| Lateral ventricle (L) | 590 | 21636.6 (9405.6) | 80 | 21130.2 (8774.9) | 69 | 20953.3 (9052.4) | 0.199 | 0.819 | 0.000 |
| Lateral ventricle (R) | 590 | 19249.9 (7985.0) | 79 | 18350.0 (7501.0) | 70 | 19229.7 (8104.4) | 0.356 | 0.701 | 0.000 |
| Third ventricle | 597 | 1873.9 (573.2) | 79 | 1925.4 (575.4) | 70 | 1938.9 (566.7) | 1.026 | 0.359 | 0.003 |
| Temporal horn of lateral ventricle (L) | 575 | 975.7 (578.4) | 79 | 942.6 (522.9) | 70 | 949.4 (488.7) | 0.016 | 0.984 | 0.000 |
| Temporal horn of lateral ventricle (R) | 577 | 920.6 (553.2) | 78 | 922.0 (571.5) | 70 | 961.8 (540.7) | 0.742 | 0.476 | 0.002 |
|  |  |  |  |  |  |  |  |  |  |
| Module 2: Corpus callosum | 571 | 3242.0 (431.4) | 76 | 3149.1 (386.2) | 68 | 3212.1 (439.1) | 2.256 | 0.106 | 0.006 |
| Anterior corpus callosum | 591 | 878.9 (142.6) | 78 | 843.4 (133.0) | 70 | 878.3 (136.8) | 3.037 | 0.049‡ | 0.008 |
| Central corpus callosum | 598 | 447.0 (106.9) | 80 | 433.4 (74.4) | 70 | 431.8 (84.1) | 1.259 | 0.284 | 0.003 |
| Middle anterior corpus callosum | 593 | 431.0 (84.2) | 80 | 423.3 (88.5) | 70 | 421.4 (94.6) | 0.962 | 0.382 | 0.003 |
| Middle posterior corpus callosum | 590 | 476.7 (110.7) | 79 | 465.4 (94.0) | 69 | 484.7 (104.7) | 0.687 | 0.504 | 0.002 |
| Posterior corpus callosum | 585 | 1011.6 (158.9) | 79 | 971.2 (163.2) | 69 | 998.2 (181.0) | 2.531 | 0.080 | 0.007 |
|  |  |  |  |  |  |  |  |  |  |
| Module 3: Limbic system | 586 | 10731.3 (1551.6) | 79 | 10589.5 (1392.8) | 69 | 10436.9 (1693.9) | 2.911 | 0.055 | 0.008 |
| Hippocampus (L) | 598 | 3441.2 (519.9) | 80 | 3441.7 (471.0) | 70 | 3410.7 (557.0) | 0.410 | 0.664 | 0.001 |
| Hippocampus (R) | 596 | 3568.4 (532.9) | 80 | 3530.8 (544.4) | 70 | 3463.2 (611.8) | 2.478 | 0.085 | 0.007 |
| Amygdala (L) | 598 | 1327.3 (281.6) | 80 | 1294.6 (273.2) | 70 | 1337.3 (305.4) | 1.581 | 0.206 | 0.004 |
| Amygdala (R) | 596 | 1538.8 (290.7) | 79 | 1511.7 (238.5) | 70 | 1527.4 (298.2) | 2.045 | 0.130 | 0.006 |
| Nucleus accumbens (L) | 595 | 418.5 (95.1) | 80 | 406.9 (83.4) | 69 | 397.0 (108.2) | 2.199 | 0.112 | 0.006 |
| Nucleus accumbens (R) | 593 | 445.3 (88.5) | 80 | 423.7 (85.3) | 69 | 430.1 (83.0) | 3.469 | 0.032‡ | 0.009 |
|  |  |  |  |  |  |  |  |  |  |
| Module 4: Diencephalon | 583 | 20154.4 (1466.9) | 80 | 19857.4 (1499.4) | 69 | 19532.7 (1428.3) | 6.589 | 0.001*† | 0.018 |
| Thalamus proper (L) | 595 | 6410.0 (577.1) | 80 | 6327.2 (572.3) | 70 | 6168.1 (571.3) | 5.792 | 0.003*† | 0.016 |
| Thalamus proper (R) | 595 | 6268.4 (562.7) | 80 | 6149.4 (537.7) | 70 | 6080.3 (513.2) | 3.951 | 0.020*† | 0.011 |
| Ventral diencephalon (L) | 590 | 3769.4 (349.8) | 80 | 3712.8 (357.5) | 69 | 3684.8 (366.2) | 3.214 | 0.041*† | 0.009 |
| Ventral diencephalon (R) | 590 | 3718.5 (337.2) | 80 | 3668.1 (350.2) | 70 | 3645.6 (338.2) | 3.033 | 0.049*† | 0.008 |
|  |  |  |  |  |  |  |  |  |  |
| Module 5: Striatum | 568 | 18859.2 (1723.8) | 76 | 18692.4 (1688.9) | 68 | 18543.3 (1876.2) | 1.642 | 0.194 | 0.005 |
| Caudate (L) | 588 | 3290.8 (421.3) | 79 | 3235.7 (365.5) | 69 | 3190.1 (406.9) | 2.442 | 0.088 | 0.007 |
| Caudate (R) | 588 | 3403.1 (438.4) | 78 | 3368.4 (439.7) | 69 | 3308.2 (430.6) | 1.631 | 0.197 | 0.004 |
| Putamen (L) | 589 | 4229.3 (528.6) | 79 | 4170.3 (498.9) | 69 | 4141.6 (587.8) | 1.502 | 0.223 | 0.004 |
| Putamen (R) | 592 | 4268.8 (550.0) | 77 | 4299.4 (507.0) | 69 | 4203.4 (620.8) | 0.747 | 0.474 | 0.002 |
| Pallidum (L) | 595 | 1883.4 (231.5) | 80 | 1864.7 (215.7) | 69 | 1875.7 (256.7) | 0.425 | 0.654 | 0.001 |
| Pallidum (R) | 594 | 1840.0 (215.0) | 79 | 1819.4 (220.1) | 69 | 1809.8 (244.8) | 1.333 | 0.264 | 0.004 |

Data are presented as means (SD). Short-duration = diabetes duration ≤ 6 years; long-duration = diabetes duration > 6 years. ANCOVA, adjusted for age, sex, ApoE-ε4 carrier status, hypertension, dyslipidemia, weight, BMI, depression scores and stroke history. L, left hemisphere; R, right hemisphere; N, number of participants; CSF, cerebrospinal fluid. **P* value was considered significant after FDR correction within each module. † mean control > long-duration. ‡ mean control > short-duration.

**Supplementary Table 5.** Post hoc analyses among the short-duration diabetes patients, long-duration diabetes patients and controls

| Region of Interest | Controls vs. Short-duration patients | | | | Controls vs. Long-duration patients | | | | | Short-duration vs. Long-duration | | | | |
| --- | --- | --- | --- | --- | --- | --- | --- | --- | --- | --- | --- | --- | --- | --- |
|  | *p value* | Partial η^2^ | *Cohen's d* | 95% CI for *Cohen's d* | | *p value* | Partial η^2^ | *Cohen's d* | 95% CI for *Cohen's d* | | *p value* | Partial η^2^ | *Cohen's d* | 95% CI for *Cohen's d* |
| Module 1: Ventricular system | 0.432 | 0.000 | 0.093 | (-0.147, 0.333) | | 0.714 | 0.000 | -0.045 | (-0.293, 0.203) | | 0.430 | 0.005 | -0.119 | (-0.445, 0.207) |
| CSF | 0.533 | 0.000 | -0.075 | (-0.310, 0.160) | | 0.37 | 0.001 | -0.115 | (-0.363, 0.133) | | 0.868 | 0.000 | -0.028 | (-0.350, 0.294) |
| Lateral ventricle (L) | 0.569 | 0.000 | 0.066 | (-0.168, 0.300) | | 0.635 | 0.000 | 0.059 | (-0.189, 0.307) | | 0.934 | 0.000 | -0.013 | (-0.335, 0.309) |
| Lateral ventricle (R) | 0.362 | 0.001 | 0.105 | (-0.130, 0.340) | | 0.833 | 0.000 | -0.026 | (-0.274, 0.222) | | 0.492 | 0.003 | -0.097 | (-0.419, 0.225) |
| Third ventricle | 0.344 | 0.001 | -0.107 | (-0.342, 0.128) | | 0.239 | 0.002 | -0.142 | (-0.390, 0.106) | | 0.678 | 0.001 | -0.065 | (-0.387, 0.257) |
| Temporal horn of lateral ventricle (L) | 0.867 | 0.000 | 0.019 | (-0.216, 0.254) | | 0.862 | 0.000 | 0.021 | (-0.227, 0.269) | | 0.708 | 0.001 | -0.056 | (-0.378, 0.266) |
| Temporal horn of lateral ventricle (R) | 0.584 | 0.000 | -0.062 | (-0.299, 0.175) | | 0.258 | 0.002 | -0.135 | (-0.383, 0.113) | | 0.426 | 0.005 | -0.120 | (-0.442, 0.202) |
|  |  |  |  |  | |  |  |  |  | |  |  |  |  |
| Module 2: Corpus callosum | 0.039 | 0.007 | 0.247 | (0.008, 0.487) | | 0.438 | 0.000 | 0.100 | (-0.151, 0.352) | | 0.206 | 0.012 | -0.205 | (-0.533, 0.123) |
| Anterior corpus callosum | 0.012 | 0.010 | 0.303 | (0.069, 0.537) | | 0.513 | 0.000 | 0.084 | (-0.164, 0.332) | | 0.131 | 0.017 | -0.253 | (-0.577, 0.071) |
| Central corpus callosum | 0.229 | 0.002 | 0.144 | (-0.090, 0.378) | | 0.196 | 0.003 | 0.166 | (-0.082, 0.414) | | 0.906 | 0.000 | -0.018 | (-0.342, 0.306) |
| Middle anterior corpus callosum | 0.294 | 0.002 | 0.119 | (-0.115, 0.353) | | 0.224 | 0.002 | 0.151 | (-0.097, 0.399) | | 0.936 | 0.000 | -0.013 | (-0.337, 0.311) |
| Middle posterior corpus callosum | 0.448 | 0.000 | 0.091 | (-0.144, 0.326) | | 0.485 | 0.000 | -0.090 | (-0.339, 0.159) | | 0.172 | 0.014 | -0.227 | (-0.551, 0.097) |
| Posterior corpus callosum | 0.027 | 0.007 | 0.270 | (0.035, 0.505) | | 0.501 | 0.000 | 0.088 | (-0.162, 0.338) | | 0.465 | 0.004 | -0.119 | (-0.443, 0.205) |
|  |  |  |  |  | |  |  |  |  | |  |  |  |  |
| Module 3: Limbic system | 0.115 | 0.004 | 0.169 | (-0.066, 0.404) | | 0.035 | 0.007 | 0.241 | (-0.009, 0.490) | | 0.547 | 0.003 | 0.088 | (-0.199, 0.411) |
| Hippocampus (L) | 0.099 | 0.004 | 0.180 | (-0.054, 0.414) | | 0.047 | 0.006 | 0.232 | (-0.015, 0.480) | | 0.639 | 0.002 | 0.070 | (-0.251, 0.391) |
| Hippocampus (R) | 0.259 | 0.002 | 0.123 | (-0.111, 0.357) | | 0.028 | 0.007 | 0.255 | (0.007, 0.503) | | 0.394 | 0.005 | 0.126 | (-0.195, 0.447) |
| Amygdala (L) | 0.077 | 0.005 | 0.195 | (-0.039, 0.429) | | 0.744 | 0.000 | 0.038 | (-0.210, 0.286) | | 0.346 | 0.006 | -0.139 | (-0.460, 0.182) |
| Amygdala (R) | 0.069 | 0.005 | 0.203 | (-0.032, 0.438) | | 0.241 | 0.002 | 0.138 | (-0.110, 0.386) | | 0.646 | 0.002 | -0.072 | (-0.393, 0.249) |
| Nucleus accumbens (L) | 0.228 | 0.002 | 0.134 | (-0.100, 0.368) | | 0.067 | 0.005 | 0.220 | (-0.028, 0.468) | | 0.506 | 0.003 | 0.107 | (-0.214, 0.428) |
| Nucleus accumbens (R) | 0.019 | 0.008 | 0.266 | (0.032, 0.500) | | 0.133 | 0.003 | 0.184 | (-0.064, 0.432) | | 0.597 | 0.002 | -0.085 | (-0.406, 0.236) |
|  |  |  |  |  | |  |  |  |  | |  |  |  |  |
| Module 4: Diencephalon | 0.079 | 0.005 | 0.194 | (-0.040, 0.428) | | <0.001* | 0.019 | 0.417 | (0.169, 0.665) | | 0.189 | 0.012 | 0.202 | (-0.121, 0.525) |
| Thalamus proper (L) | 0.224 | 0.002 | 0.137 | (-0.097, 0.371) | | <0.001* | 0.017 | 0.406 | (0.158, 0.654) | | 0.098 | 0.020 | 0.266 | (-0.057, 0.589) |
| Thalamus proper (R) | 0.133 | 0.003 | 0.171 | (-0.063, 0.405) | | 0.009* | 0.011 | 0.323 | (0.075, 0.571) | | 0.406 | 0.005 | 0.132 | (-0.191, 0.455) |
| Ventral diencephalon (L) | 0.091 | 0.004 | 0.195 | (-0.039, 0.429) | | 0.025* | 0.008 | 0.279 | (0.031, 0.527) | | 0.737 | 0.000 | 0.050 | (-0.273, 0.373) |
| Ventral diencephalon (R) | 0.111 | 0.004 | 0.182 | (-0.052, 0.416) | | 0.030* | 0.007 | 0.265 | (0.017, 0.513) | | 0.769 | 0.000 | 0.045 | (-0.278, 0.368) |
|  |  |  |  |  | |  |  |  |  | |  |  |  |  |
| Module 5: Striatum | 0.430 | 0.000 | 0.096 | (-0.098, 0.330) | | 0.080 | 0.005 | 0.225 | (-0.023, 0.473) | | 0.659 | 0.001 | 0.073 | (-0.255, 0.401) |
| Caudate (L) | 0.197 | 0.003 | 0.159 | (-0.075, 0.393) | | 0.045 | 0.006 | 0.265 | (0.017, 0.513) | | 0.493 | 0.003 | 0.115 | (-0.208, 0.438) |
| Caudate (R) | 0.404 | 0.001 | 0.103 | (-0.131, 0.337) | | 0.064 | 0.005 | 0.244 | (-0.005, 0.492) | | 0.430 | 0.005 | 0.132 | (-0.191, 0.455) |
| Putamen (L) | 0.365 | 0.001 | 0.106 | (-0.234, 0.340) | | 0.113 | 0.004 | 0.199 | (-0.049, 0.447) | | 0.661 | 0.001 | 0.070 | (-0.253, 0.393) |
| Putamen (R) | 0.683 | 0.000 | -0.049 | (-0.283, 0.185) | | 0.290 | 0.002 | 0.134 | (-0.114, 0.382) | | 0.296 | 0.008 | 0.171 | (-0.152, 0.494) |
| Pallidum (L) | 0.439 | 0.000 | 0.092 | (-0.142, 0.326) | | 0.524 | 0.000 | 0.082 | (-0.166, 0.330) | | 0.687 | 0.001 | -0.067 | (-0.390, 0.256) |
| Pallidum (R) | 0.345 | 0.001 | 0.114 | (-0.120, 0.348) | | 0.138 | 0.003 | 0.193 | (-0.055, 0.441) | | 0.938 | 0.000 | 0.013 | (-0.310, 0.336) |

Short-duration = diabetes duration ≤ 6 years; long-duration = diabetes duration > 6 years. ANCOVA, adjusted for age, sex, ApoE-ε4 carrier status, hypertension, dyslipidemia, weight, BMI, depression scores and stroke history. L, left hemisphere; R, right hemisphere; CSF, cerebrospinal fluid. **P* value was considered significant after FDR correction within each module.
